# Organizing Care Matters: Fragmented Pathways Double Early Local Recurrence Risk in Sarcoma

**DOI:** 10.3390/cancers18030387

**Published:** 2026-01-27

**Authors:** Markus Schärer, Philip Heesen, Gabriela Studer, Bettina Vogel, Bruno Fuchs, on behalf of the Swiss Sarcoma Network

**Affiliations:** 1 Sarcoma Service, Department of Orthopedics and Trauma, Kantonsspital Winterthur, 8400 Winterthur, Switzerland; 2 Department of Shoulder and Elbow Surgery, Schulthess Clinic Zurich, 8008 Zurich, Switzerland; 3 Medical Faculty, University of Zurich, 8032 Zurich, Switzerland; 4 Faculty of Health Sciences & Medicine, University of Lucerne, Frohburgstrasse 3, 6002 Luzern, Switzerland; 5 Sarcoma Service, Department of Radiation Oncology, Sarcoma Center, LUKS University Hospital, 6000 Lucerne, Switzerland; 6 Sarcoma Service, Department of Orthopedics and Trauma, Sarcoma Center, LUKS University Hospital, 6000 Lucerne, Switzerland

**Keywords:** sarcoma, local recurrence, care pathway, fragmented care, unplanned “whoops” excision, surgical margins, multidisciplinary care, real-world data

## Abstract

**Background**: Early local recurrence (ELR) in musculoskeletal sarcoma is associated with poor oncologic outcomes, yet the relative impact of tumor biology versus system-level factors remains insufficiently understood. This multicenter real-world study within the Swiss Sarcoma Network evaluated whether the initial care pathway influences the risk and timing of ELR. **Methods**: Patients with histologically confirmed sarcoma and documented local recurrence were classified according to initial management within a Comprehensive Care Pathway (CCP) or a Fragmented Care Pathway (FCP). ELR was defined as recurrence within 12 months after index surgery. Associations were analyzed using restricted Cox proportional hazards models and Firth-penalized logistic regression, adjusting for key clinicopathologic factors. Follow-up was calculated from index surgery to death or administrative censoring (median 88.2 months; interquartile range, 54.9–141.6). **Results**: Among 158 patients with local recurrence, 96 (60.8%) were treated within CCP, and 62 (39.2%) entered through FCP. ELR occurred in 53 patients (33.5%) and was more frequent in the FCP cohort. Fragmented care was independently associated with ELR in both time-to-event analysis (hazard ratio 2.00, 95% CI 1.14–3.51) and penalized logistic regression (odds ratio 2.83, 95% CI 1.09–6.94). Unplanned (“whoops”) procedures and incomplete resection margins were substantially more common in FCP and independently predicted ELR. Tumor grade also contributed to risk, but the magnitude of the pathway effect was comparable. ELR was associated with higher rates of synchronous metastases and inferior survival compared with late local recurrence. Adjuvant therapy did not independently reduce ELR risk after adjustment for surgical quality. **Conclusions**: These findings indicate that ELR in musculoskeletal sarcoma is strongly influenced by modifiable system-level factors. Early referral, multidisciplinary evaluation, and expert margin-oriented surgery are critical to reducing early recurrence and improving patient outcomes.

## Simple Summary

Sarcomas are rare malignancies in which outcomes strongly depend on early management according to established guidelines in specialized centers. Nevertheless, many patients receive initial treatment outside structured sarcoma care pathways, where diagnostic and surgical standards are often not fully met. In this study, we analyzed patients with local recurrence within the Swiss Sarcoma Network to assess how the initial care pathway influences the risk of early recurrence. We found that fragmented initial management was independently associated with a higher risk of early local recurrence, mainly due to unplanned surgery and incomplete tumor removal. This increased risk was not compensated for by adjuvant treatments. Our findings highlight the importance of early referral and coordinated, center-based care to improve outcomes in patients with musculoskeletal sarcoma.

## 1. Introduction

Sarcomas are rare, heterogeneous malignant tumors whose nonspecific clinical presentation frequently overlaps with benign conditions, leading to delayed recognition and misdiagnosis—particularly in non-specialized settings where clinicians encounter these tumors infrequently [1,2]. Consequently, many patients undergo initial treatment outside dedicated sarcoma centers, often without guideline-directed imaging, biopsy, or oncologic preoperative planning [3]. This fragmented entry into care is associated with unplanned “whoops” resections -excisions performed without oncologic work-up—and with inadequate surgical margins, both of which are linked to residual disease and inferior local control [4,5].

Centralized care within specialized sarcoma centers improves diagnostic accuracy, treatment planning, and oncologic decision-making through multidisciplinary tumor boards [6], experienced sarcoma surgeons, dedicated musculoskeletal radiology and pathology, and coordinated radiotherapy and systemic treatment [7]. Hub-and-spoke referral models aim to triage suspected sarcomas so that staging, biopsy strategy, and definitive surgery are guided from the outset [8,9]. Nonetheless, real-world fragmentation remains frequent, driven by delayed recognition, geographic access, and non-standardized referral, and has been associated with higher local recurrence (LR) and suboptimal treatment sequences [10,11].

LR is a central measure of surgical and oncologic quality in soft tissue sarcoma [12,13]. While tumor grade is an established biological determinant of recurrence, surgical quality—particularly the avoidance of unplanned excisions and the achievement of negative (R0) margins—plays a critical role in local control [14,15]. Importantly, early local recurrence (ELR), defined as recurrence within 12 months of index surgery, appears clinically aggressive [16]: ELR has been associated with greater spread and poorer survival, indicating that early failures are not merely a matter of timing but reflect adverse disease-care interactions.

Despite international guidelines emphasizing early referral and multidisciplinary management, real-world evaluations that explicitly link care pathways to ELR and test whether this association is mediated by surgical-quality intermediates (whoops resections, margin status) are limited [17]. Switzerland’s decentralized delivery system and the establishment of the Swiss Sarcoma Network (SSN) provide a pragmatic framework to examine how patients enter the sarcoma pathway, how this affects surgical quality, and how these elements together influence the development and timing of LR within a national network [7,18].

Herein, we tested whether fragmented initial management—particularly treatment outside the sarcoma network—is associated with an increased risk of early recurrence compared with comprehensive center-based care. We evaluated the relationships among care pathway, unplanned resection, and surgical margin status in relation to recurrence timing, and we examined how ELR correlates with metastatic disease at diagnosis and survival outcomes [19,20]. By distinguishing pathway-related determinants from intrinsic tumor biology, this work aims to inform system-level quality improvement and underscore early multidisciplinary triage and expert, margin-oriented surgery as key levers to prevent early local failure.

## 2. Materials and Methods

### 2.1. Study Design and SSN

This study used prospective real-world-time data (RWTD) from the SSN-ShapeHub^®^ registry, established in 2018 as a national interoperable data warehouse for quality monitoring in sarcoma care [21]. The registry consolidates information discussed during weekly Multidisciplinary Team/Sarcoma Board (MDT/SB) meetings, capturing care pathways, surgical practices, and outcomes across participating centers. These MDT/SB meetings ensure continuous data validation, standardized documentation, and transdisciplinary decision-making, providing a comprehensive dataset to evaluate the impact of care fragmentation on surgical quality and LR. Switzerland’s decentralized, open-referral healthcare delivery allows patients to undergo diagnostic evaluation and initial treatment outside specialized sarcoma networks prior to expert review. Within this context, we defined the Fragmented Care Pathway (FCP) as initial management outside the sarcoma network, characterized by delayed or non–guideline-concordant sequencing of staging, biopsy, and surgery. In contrast, the Comprehensive Care Pathway (CCP) denotes network-coordinated entry with early multidisciplinary triage, guideline-concordant staging and biopsy-first strategies, followed by center-based surgery within the SSN. This distinction reflects process organization and care sequencing, rather than tumor histology or biological characteristics.

### 2.2. Subjects and Data Extraction

All consecutive patients presented to the SSN MDT/SB between 2018 and 2025 were eligible, including those with suspected sarcoma and secondary referrals after initial management in external institutions. Patients were analyzed based on the date of first histologically confirmed sarcoma diagnosis and the occurrence of their first LR. As patients referred from outside institutions could have been diagnosed before entering the SSN, diagnostic dates were allowed to precede the study period. All patients treated within the SSN during 2018–2025 were followed for LR within the same timeframe. Data were extracted using the Adjumed platform (Adjumed Services AG, Zurich, Switzerland; accessed December 2025), including referral patterns, treatment modalities, and surgical outcomes.

### 2.3. Definitions, Outcomes, Measurements, and Clinical Characteristics

Demographic and treatment-related variables were obtained from the RWTD system. All diagnoses were established by sarcoma reference pathologists using an integrated approach (morphology and immunohistochemistry, with histotype-contingent molecular testing when indicated) within MDT review. The study was not designed to evaluate molecular correlates of local recurrence. Key pathway-related variables included unplanned “whoops” resections, treatment pathway category (comprehensive care pathway, CCP, within the SSN; or fragmented care pathway, FCP, indicating initial external treatment), and adjuvant therapies. Tumor-related data included anatomic site (extremity, abdomen/retroperitoneum, axial, head and neck, urogenital/perineal/anal), compartment classification (superficial soft tissue, deep soft tissue, bone sarcomas), and size categories (0–50 mm, 51–100 mm, 101–150 mm, >150 mm) [22]. Margins were documented according to Enneking et al. [23] and Gundle et al. [13] (R0, R1, R2). Tumor grading followed the Angervall and Kindblom system [24] (G1–G3). Local recurrence was classified as early local recurrence (ELR) if it occurred within 12 months after the index surgery, and as late local recurrence (LLR) if it occurred more than 12 months after the index surgery. Overall survival and metastatic status are reported descriptively to contextualize ELR; this study was not designed to model the causal interplay between LR timing and metastasis. The cohort reflects musculoskeletal sarcomas dominated by soft-tissue tumors; a small number of bone sarcomas were retained because the organizational mechanism under study (entry pathway **→** surgical quality) is shared across domains.

### 2.4. Statistical Analysis

Descriptive statistics were used to summarize patient demographics, tumor characteristics, treatment modalities, and care pathways. Categorical variables were compared using the Chi-Square test or Fisher’s exact test when expected cell counts were low. Standardized mean differences (SMDs) were calculated to quantify imbalances between the comprehensive care pathway and fragmented care pathway. Timing of local recurrence was evaluated using empirical cumulative distribution functions and visualized with stratified curves. Given limited ELR events, we pre-specified a minimal cause-specific Cox model for the first 12 months, including two a priori covariates—care pathway (system factor of interest) and tumor grade (biologic determinant)—to respect events-per-variable constraints. Because of quasi-separation and sparse cells, we complemented this with a Firth-penalized multivariable logistic regression for ELR (yes/no). Effect sizes are reported with 95% CIs; inference is limited to the first-year window. Because of the limited number of ELR events and evidence of quasi-separation for several predictors, a Firth-penalized logistic regression was conducted as a sensitivity analysis to obtain bias-reduced, stable estimates. Hazard ratios (HRs), odds ratios (ORs), and 95% confidence intervals (CIs) were reported for all models. All analyses were performed using SAS version 9.4 (SAS Institute, Cary, NC, USA). Conceptualization of the analytical strategy was supported through interaction with ChatGPT 5.2 thinking (OpenAI), last accessed on December 2025. Statistical significance was defined as *p* < 0.05. Within the 12-month estimand, proportional-hazards assumptions were reviewed; no material violations were detected for prespecified covariates.

## 3. Results

### 3.1. Study Patient Population

**Figure 1:**
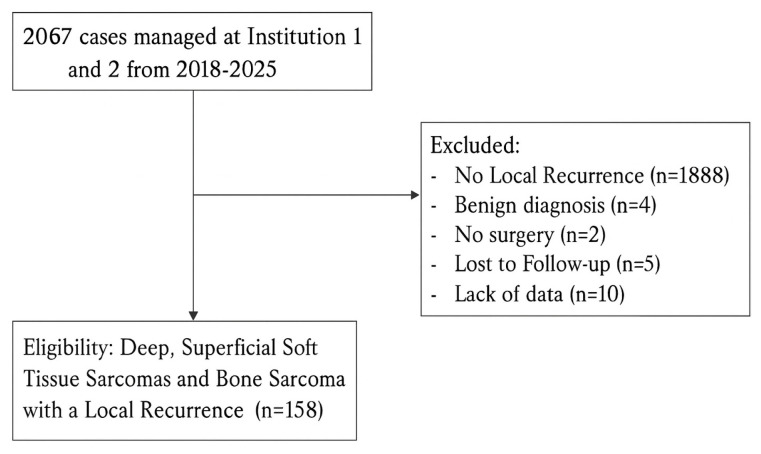
Flowchart of patient selection showing inclusion of 158 cases with LR after applying eligibility and exclusion criteria.

Over an 8-year period (2018–2025), a total of 2067 patients with suspected sarcoma were presented to the MDT-SB of the SSN. Among these, 158 patients who developed a local recurrence were included in the present analysis (Figure 1). Data were collected from two tertiary sarcoma centers, with Institution 1 managing 45.6% of cases and Institution 2, 54.4%. Major histologic subtypes represented included undifferentiated pleomorphic sarcoma, myxofibrosarcoma, liposarcoma subtypes, leiomyosarcoma, and synovial sarcoma (documentation varied across centers and calendar time). To avoid sparse-strata instability, multivariable modeling prespecified grade as the biologic anchor; the pathway–quality findings were directionally unchanged in qualitative checks within the common subtypes. Diagnoses were confirmed by expert sarcoma pathology within MDT pathways; molecular assays were performed when indicated by histotype.

**Figure 2:**
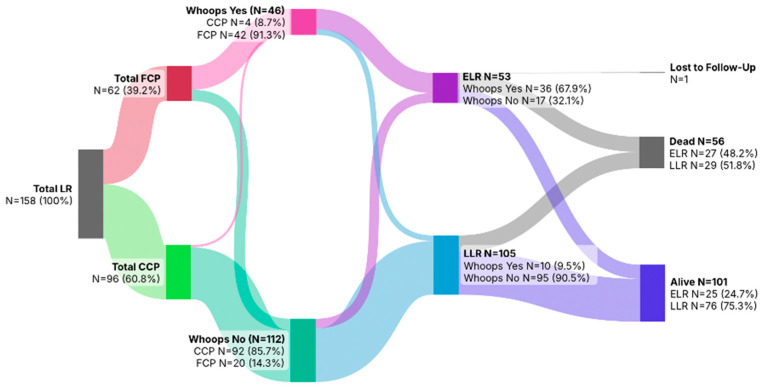
Sankey diagram showing initial care pathway, occurrence of whoops resections, timing of local recurrence (ELR/LLR), and survival outcomes among the 158 patients with local recurrence.

The Sankey diagram (Figure 2) illustrates the flow of patients with local recurrence (LR; *N* = 158) according to initial treatment setting, occurrence of an unplanned (“whoops”) procedure, and survival outcome. Primary treatment was performed entirely within a sarcoma center in 96 patients (CCP; 60.8%), whereas 62 patients (39.2%) were referred after initial treatment elsewhere (FCP). Overall, 46 patients (29.1%) underwent a whoops procedure, the vast majority of whom originated from the FCP group (*N* = 42, 91.3%), while 112 patients (70.9%) had no unplanned surgery. Local recurrences were classified as early (ELR; *N* = 53, 33.5%) or late (LLR; *N* = 105, 66.5%). ELR occurred predominantly among patients with a whoops procedure (*N* = 36, 67.9%), whereas LLR was more common in patients without unplanned surgery (*N* = 95, 90.5%). Follow-up was calculated from the date of index surgery to death or administrative censoring in December 2025. The median follow-up of the cohort was 88.2 months (interquartile range [IQR], 54.9–141.6 months). At last follow-up, 56 patients (35.4%) had died (ELR: *N* = 27, 48.2%; LLR: *N* = 29, 51.8%), 101 patients (63.9%) were alive (ELR: *N* = 25, 24.7%; LLR: *N* = 76, 75.3%), and 1 patient (0.6%) was lost to follow-up.

### 3.2. Timing of Local Recurrence by Care Pathway

**Figure 3:**
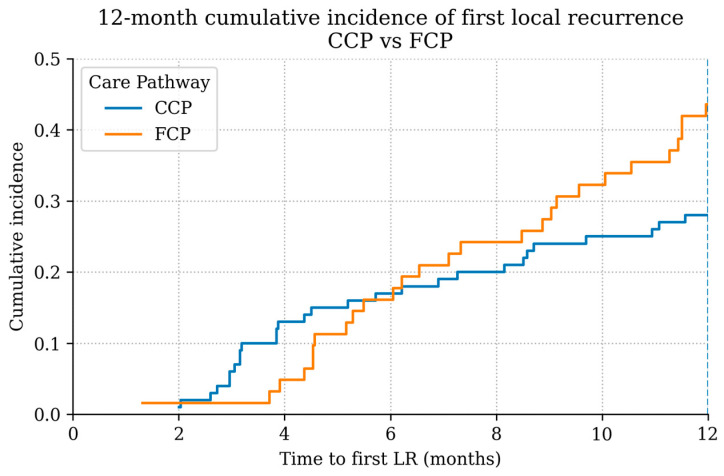
Time-to-LR distribution among LR patients by pathway (12-month landmark shown). Descriptive display; not a cumulative-incidence function.

When comparing the timing of LR between care pathways, patients initially treated in the FCP exhibited a noticeably earlier pattern of recurrence compared with those managed within the CCP (Figure 3). Among LR patients, the curve for the FCP group rose more steeply during the early follow-up period, indicating that a greater proportion of LR occurred shortly after primary treatment. In contrast, recurrence among CCP patients accumulated more gradually over time.

### 3.3. Time to Local Recurrence by Tumor Site

**Figure 4:**
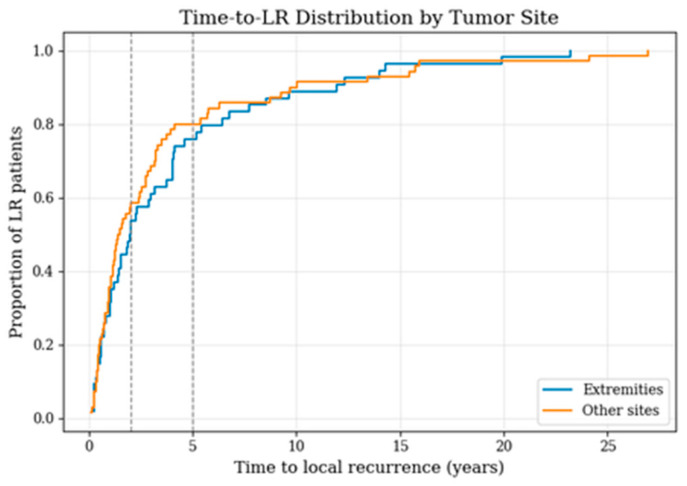
Time-to-local-recurrence distribution among patients with LR, comparing extremity sarcomas with tumors at other sites; vertical lines indicate the 2- and 5-year time points.

A similar analysis was performed to evaluate whether the timing of recurrence differed by tumor site (Table 1; Figure 4). Due to low case numbers in non-extremity locations, these anatomical sites were merged into a single “other sites” category to allow for a more robust and comparable analysis. Among LR patients with extremity tumors (*n* = 64), 54.7% experienced recurrence within two years and 78.1% within five years. In comparison, LR patients with tumors located at other anatomical sites (*n* = 94) showed slightly higher proportions of earlier recurrence, with 61.3% relapsing by two years and 82.8% by five years. The empirical cumulative distribution curves illustrate this pattern, with non-extremity tumors displaying a steeper early increase.

### 3.4. Characteristics and Factors Associated with Early Local Recurrence

In the present cohort (Table 2), several parameters differed significantly between patients treated within the CCP and those managed in the FCP. Deep musculoskeletal sarcomas were more common in the FCP group (19.5% vs. 5.2%, *p* = 0.005). Tumor grading also varied significantly between groups (*p* = 0.004), with high-grade sarcomas (G3) more frequent in the CCP cohort (61.5% vs. 28.4%) and low-grade tumors (G1) more common in the FCP group (33.3% vs. 13.5%).

Surgical quality indicators showed marked differences. Incomplete resections (R1/R2) occurred more frequently in the FCP group (70.2% vs. 47.6%, *p* = 0.002), and unplanned (“whoops”) resections were significantly more common in the FCP cohort (67.8% vs. 4.2%, *p* < 0.001). Adjuvant treatment also differed between pathways (*p* = 0.02), 54.2% of CCP patients received adjuvant therapy compared with 32.2% of FCP patients.

Local recurrence dynamics varied between groups. Early local recurrence (ELR) occurred more often in the FCP cohort (36.1% vs. 31.4%, *p* = 0.05).

In the comparative analysis of ELR and LLR, two factors showed statistically significant differences. Patients with ELR more frequently presented with synchronous metastases at diagnosis (15.1% vs. 4.8%, *p* = 0.02) and had lower survival at last follow-up (48.1% vs. 72.4%, *p* = 0.002). Tumor grading demonstrated a non-significant trend toward higher-grade lesions in the ELR group (G3: 58.5% vs. 49.5%, *p* = 0.09). Margin status and adjuvant therapy did not differ significantly between ELR and LLR. Unplanned resections were substantially more common in the ELR cohort (67.9% vs. 9.5%).

LR occurred most frequently in intra- and retroperitoneal sarcomas (26.6%), followed by axial locations (25.9%), upper extremity tumors (22.2%), and lower extremity tumors (18.3%), whereas head and neck (5.1%) and urogenital/perianal locations (1.9%) were less commonly affected. When stratified by care pathway, extremity sarcomas accounted for a higher proportion of LR in the CCP group (upper and lower extremity combined: 42.7%) compared with the FCP group (37.1%), whereas axial and intra-abdominal locations were relatively more frequent among FCP patients. Overall, 64 recurrences arose in extremity sarcomas and 94 in non-extremity locations.

### 3.5. Univariable and Multivariable Cox Proportional Hazard Analysis

In the univariable Cox proportional hazards analysis (Table 3), none of the assessed variables reached statistical significance for predicting ELR. Treatment institution showed no association with recurrence risk (HR 1.11, 95% CI 0.65–1.92, *p* = 0.698). Tumor grading demonstrated a trend toward higher ELR risk with increasing grade, although this did not reach significance (HR 1.40, 95% CI 0.98–2.01, *p* = 0.066). Tumor localization, unplanned excision, tumor size, and resection status were all unrelated to ELR in this analysis (all *p* > 0.3). The care pathway also showed a non-significant trend toward higher recurrence among patients treated outside the structured pathway (HR 1.58, 95% CI 0.92–2.71, *p* = 0.097). Although not statistically significant, the effect sizes for tumor grading and care pathway suggested potential clinical relevance, supporting their inclusion in subsequent multivariable modeling.

**Figure 5:**
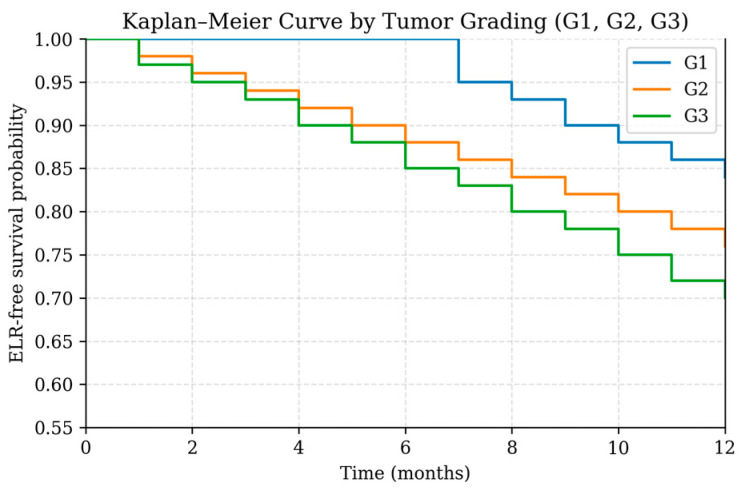
Kaplan–Meier curve showing early local recurrence (ELR)-free survival stratified by tumor grading (G1, G2, G3) during the first 12 months after treatment.

**Figure 6:**
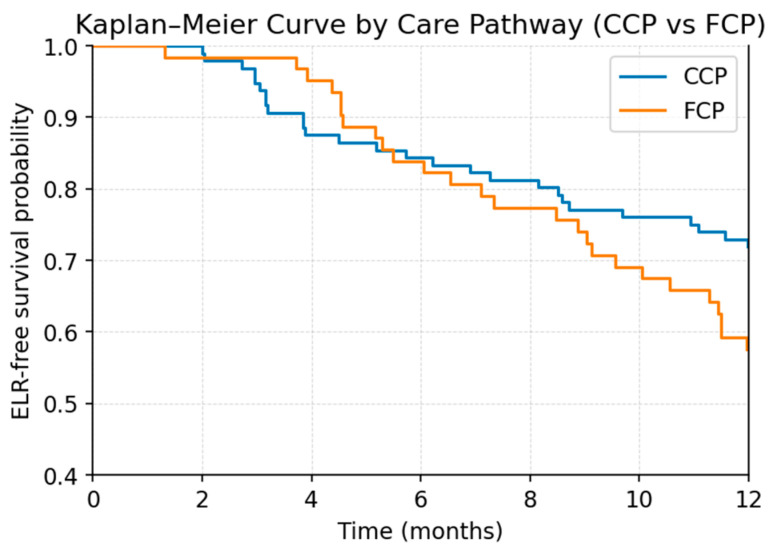
Kaplan–Meier curve showing early local recurrence (ELR)-free survival stratified by care pathway (comprehensive care pathway, CCP; fragmented care pathway, FCP) during the first 12 months after treatment.

Because of the limited number of events (ELR, n = 53), the multivariable Cox proportional hazards model was intentionally restricted to two key predictors—tumor grading and care pathway—that demonstrated the strongest signals in univariable analysis. In this focused model (Table 4; Figure 5 and Figure 6), both variables emerged as statistically significant independent predictors of ELR. Higher tumor grade was associated with an increased hazard of recurrence (HR 1.59, 95% CI 1.09–2.31, *p* = 0.0149). Likewise, patients treated outside the coordinated care pathway demonstrated a significantly higher risk of ELR compared with those managed within the structured pathway (HR 2.00, 95% CI 1.14–3.51, *p* = 0.0150).

### 3.6. Sensitivity Analysis of the Early Local Recurrence Cut-Off

As a sensitivity analysis, the definition of ELR was extended from 12 to 18 months after index surgery. Using the 18-month cut-off, early recurrence remained significantly more frequent in the FCP than in the CCP (53.2% vs. 32.3%; χ^2^ *p* = 0.014). In a restricted Cox proportional hazards model including tumor grade and care pathway, treatment within the FCP continued to predict earlier recurrence (HR 1.74, 95% CI 1.09–2.77; *p* = 0.0206), while higher tumor grade remained independently associated with recurrence timing (HR 1.56, 95% CI 1.15–2.12; *p* = 0.0043).

### 3.7. Firth-Penalized Logistic Regression for Early Local Recurrence

Because several variables showed low event counts and indications of separation in the univariable and multivariable Cox analyses, we performed an additional Firth-penalized logistic regression as a sensitivity analysis. This method reduces small-sample bias and provides stable, bias-corrected estimates when standard regression models become unreliable. The Firth model allowed us to confirm the robustness of key predictors identified in the Cox analysis, particularly for variables with sparse categories or imbalanced event distributions.

**Figure 7:**
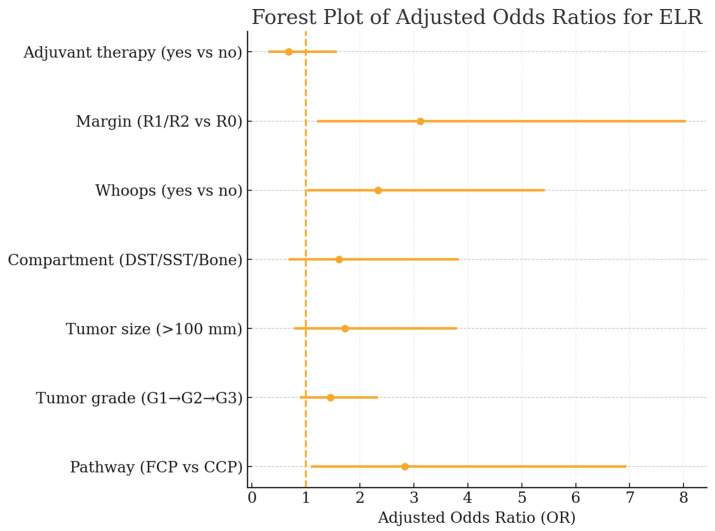
Forest plot of adjusted odds ratios (OR) from the multivariable Firth-penalized logistic regression model identifying independent predictors of early local recurrence. Displayed are ORs with corresponding 95% confidence intervals for care pathway, tumor grade, tumor size, anatomical compartment, unplanned (“whoops”) resections, resection margin status, and adjuvant therapy.

In the multivariable Firth-penalized logistic regression model for ELR (Table 5, Figure 7), treatment in an FCP remained a significant independent predictor of early relapse (OR 2.83, 95% CI 1.09–6.94, *p* = 0.031). Incomplete resections (R1/R2) and unplanned excisions were likewise associated with a substantially increased ELR risk (OR 3.12, *p* = 0.019 and OR 2.34, *p* = 0.046, respectively). Higher tumor grade and larger size showed non-significant trends toward elevated risk, whereas adjuvant radiotherapy did not independently modify ELR likelihood.

### 3.8. Surgical Quality and Adjuvant Therapy by Care Pathway

Marked differences in surgical quality indicators and adjuvant treatment use were observed between the CCP and the FCP (Table 6). Whoops procedures were dramatically more frequent in the FCP cohort (68.9%) compared with the CCP cohort (4.2%, *p* < 0.001), corresponding to a very large standardized difference (SMD = −1.42). Incomplete resections (R1/R2) also occurred more often in the FCP group (70.2%) than in the CCP group (47.6%, *p* = 0.002), generating a moderate-to-large imbalance (SMD = −0.45). Use of adjuvant therapy differed meaningfully between pathways: 54.2% of CCP patients received radiotherapy, chemotherapy, or combined therapy compared with 32.8% of FCP patients (*p* = 0.020; SMD = +0.43). Data on post-whoops re-excision and definitive margins were variably recorded and incomplete; counts are therefore reported descriptively (where available) and not used for inferential modeling.

## 4. Discussion

In this multicenter real-world analysis within the SSN, early local recurrence in sarcoma emerged as a phenomenon shaped not only by tumor biology but also—critically—by the organization and quality of initial care. Fragmented entry into the system was strongly and independently associated with ELR in both the restricted Cox model (FCP HR 2.00, 95% CI 1.14–3.51) and the Firth-penalized model (OR 2.83, 1.09–6.94). These findings are concordant with international reports linking deviations from guideline-concordant diagnostic pathways to unplanned surgery and compromised oncologic safety [22,23,24]. Together, our data delineate a plausible and modifiable sequence—care pathway → surgical quality (whoops/margins) → ELR → survival—which is summarized schematically in Figure S1, underscoring the central importance of early multidisciplinary triage and specialized sarcoma care. Because the pathway–quality mechanism targets organizational steps (early MDT triage, biopsy-first sequencing, margin-oriented surgery), it is transferable to both open-referral systems and gatekept or centralized healthcare models that aim to prevent unplanned excisions and shorten preoperative timelines; differences in referral architecture may modify the magnitude, but not the direction, of the effect. These organizational levers apply across musculoskeletal sarcomas, supporting the generalizability of the pathway–quality mechanism.

FCP was one of the strongest independent predictors of ELR across both modeling approaches. Patients managed initially outside the network exhibited earlier recurrence dynamics compared with those entering through the Comprehensive Care Pathway. This aligns with network/registry observations that initial care outside specialist centers is associated with inferior control and higher reoperation rates [8,25,26]. Notably, the magnitude of the pathway effect in our study rivals or exceeds key biological parameters, highlighting system-level determinants that are actionable through structural interventions such as mandatory early referral, standardized diagnostic algorithms, and MDT triage.

The observed early peak of local recurrence within the CCP—particularly during the first three months after index surgery—likely reflects referral and selection bias rather than inferior care quality. In our cohort, patients treated primarily within the sarcoma center presented significantly more often with biologically aggressive tumors (G3: 61.5% in CCP vs. 28.4% in FCP, *p* = 0.004). This overrepresentation of high-grade disease suggests that very aggressive tumor biology itself contributes to the early rise in recurrence risk, leading to very early failures despite guideline-concordant staging, multidisciplinary planning, and high-quality, center-based treatment.

Crucially, this early CCP peak differs fundamentally from the recurrence pattern observed in the fragmented care pathway. While the CCP curve shows an initial rise followed by a flatter course—consistent with biology-driven early events—the risk of recurrence in the FCP increases continuously over the first 12 months, resulting in a higher cumulative incidence of ELR overall (36.1% vs. 31.4%, *p* = 0.05). This temporal pattern suggests that ELR in the FCP is not driven by isolated early failures, but rather by a cumulative process effect.

This interpretation is supported by multivariable analyses, in which the care pathway remained an independent predictor of ELR after adjustment for tumor grade, both in the restricted Cox model (HR 2.00, 95% CI 1.14–3.51, *p* = 0.015) and in the Firth-penalized logistic regression (OR 2.83, 95% CI 1.09–6.94, *p* = 0.031). Taken together, these findings argue against tumor biology alone as an explanation and instead support the presence of a genuine, modifiable system-level effect. Conceptually, very early recurrences within the CCP appear predominantly biology-driven, whereas early recurrences in the FCP accumulate over time and are largely mediated by preventable process failures, including markedly higher rates of unplanned (“whoops”) resections (67.8% vs. 4.2%) and incomplete margins (R1/R2: 70.2% vs. 47.6%). Importantly, this association proved robust in sensitivity analyses applying an extended 18-month definition of early local recurrence. Under this alternative cut-off, fragmented care remained significantly associated with earlier recurrence, and the care pathway continued to predict recurrence timing independently of tumor grade, supporting that the observed pathway effect is not driven by an arbitrary temporal threshold.

Mechanistically, the pathway effect is plausibly mediated by unplanned (“whoops”) excisions and incomplete (R1/R2) margins. In our LR cohort, whoops procedures were far more frequent in FCP than in CCP (68.9% vs. 4.2%), and incomplete margins were more common (70.2% vs. 47.6%). In the Firth model, both whoops and R1/R2 remained independent WLR predictors (OR 2.34 and 3.12, respectively), consistent with the literature showing that unplanned surgery and compromised margins drive residual disease and early local failure [22,24,25]. The data reinforce that ELR prevention depends foremost on correct staging, biopsy, MDT planning, and expert, margin-oriented surgery [25,26].

Tumor grade independently predicted ELR (Cox HR 1.59, 95% CI 1.09–2.31), in line with prior work indicating more aggressive local behavior in high-grade STS [26]. However, in our dataset, the pathway effect is at least as large as grade and uniquely modifiable. This balance supports the view that centralized pathways can partially offset adverse biology by preventing process failures that precipitate early recurrence [8,9].

ELR was associated with more synchronous metastases at diagnosis (15.1% vs. 4.8%) and inferior survival (alive 48.1% vs. 72.4%) compared with LLR, consistent with prior evidence demonstrating that early recurrence is a clinically malignant event and a harbinger of systemic disease. von Konow et al. reported that late local recurrences (LLR) are more salvageable and carry a better prognosis, whereas ELR is associated with significantly inferior survival [27]. This pattern affirms that ELR is clinically malignant rather than a mere timing label, and that preventing ELR is a patient-relevant objective with implications for systemic outcomes. While ELR aligned with higher metastatic burden and inferior survival in this cohort, the causal relationship between LR timing and metastasis risk was not modeled here and is the subject of a separate, dedicated analysis [28].

Patients treated within CCP were more likely to receive any adjuvant therapy (RT and/or chemotherapy); 54.2% vs. 32.8%, reflecting guideline-concordant workflows. Nevertheless, adjuvant therapy did not independently protect against ELR after adjustment for pathway and surgical quality, echoing evidence that RT and chemotherapy cannot compensate for inadequate surgery but should reinforce a properly executed, margin-oriented operative strategy [29]. The role of secondary surgical interventions following unplanned excision, including margin conversion and their potential impact on local control, was not analyzed in the present study and represents an important subject for future, dedicated investigations [30].

The preventable component of early local failure is process-driven. Systems should: (i) enact early MDT triage for any mass suspicious for STS; (ii) require stating and biopsy before any excision; (iii) channel patients to center-based, R0-oriented surgery; and (iv) standardize documentation to avoid re-excision delays. International sarcoma networks, including the French NETSARC initiative and global hub-and-spoke models, have demonstrated improved outcomes through centralized expertise, standardized evaluation, and avoidance of unplanned procedures [8,9,31]. At policy level, pathway adherence -monitored via whoops and margin KPIs- should be embedded in referral agreements and funding/quality frameworks.

Strengths include a prospective real-world-time network cohort; prespecified modeling adapted to limited events per variable (restricted Cox regression; Firth logistic); and granular capture of pathway and surgical quality variables. Limitations are intrinsic to the dataset: (i) competing-risk cumulative incidence of first LR by pathway/site could not be estimated due to incomplete competing-event timestamps; Quantifying the effect of extended resections and margin conversion would require complete timestamps and time-dependent or multi-state modeling with adequate event counts. These conditions were not met; consequently, secondary surgeries were not analyzed inferentially in the present study. A prospective, fully timestamped design is warranted to estimate margin-conversion effects on ELR. (ii) timing displays by pathway and site are descriptive among LR patients and must not be interpreted as population incidence; (iii) modest ELR event counts constrained covariate breadth in time-to-event models; and (iv) observational design leaves residual confounding despite SMDs and penalized modelling; (v) Histologic subtype documentation was heterogeneous across centers and time; therefore grade was used as the biologic anchor in multivariable models, and residual subtype confounding is possible. In addition, histologic heterogeneity (including a small number of bone sarcomas) remains; while the pathway–quality mechanism is shared across sarcoma domains, this residual heterogeneity may attenuate precision. Molecular characterization was heterogeneously available across centers and histotypes during the accrual period and was therefore not modeled. Given our systems-focused estimand (pathway → surgical quality → ELR), incomplete molecular capture is unlikely to bias the principal association; nonetheless, upcoming registry iterations will prospectively structure histotype-contingent molecular fields to enhance future analyses. A further limitation relates to the ascertainment of FCP patients. By design, patients initially treated outside the network only became observable upon referral to the SSN. Consequently, patients who experienced local recurrence and were managed entirely at external institutions without subsequent network referral—or who died before referral—could not be captured in this analysis. This differential ascertainment may introduce selection bias, as the FCP cohort represents only those patients who survived long enough and were deemed appropriate for tertiary referral. However, this bias would likely attenuate rather than inflate the observed pathway effect, as exclusion of the most unfavorable FCP outcomes would tend to make fragmented care appear more favorable than it truly is. Nevertheless, the magnitude of the true association between care fragmentation and early local recurrence may differ from our estimates, and our findings should be interpreted with this limitation in mind. These constraints inform a prospective, fully timestamped follow-up to deliver pathway- and site-specific cumulative incidence and formal mediation.

## 5. Conclusions

ELR in musculoskeletal sarcoma reflects an interplay between tumor biology and how/where patients first enter care. Fragmented initial management is associated with increased ELR risk, largely via preventable surgical-quality deficits (unplanned excisions) and inadequate margins. Because ELR coincides with worse systemic outcomes, the most effective modifiable strategies include early MDT involvement, correct diagnostic sequencing, and expert R0 surgery. Strengthening referral structures and enforcing centralized sarcoma management should be prioritized.

## Figures and Tables

**Table 1:** Timing of local recurrence stratified by tumor site. Among patients with LR, extremity sarcomas showed slightly lower proportions of recurrence within 2 and 5 years compared with sarcomas arising at other anatomical sites.

Site	N (LR Patients)	Recurred ≤ 2 Years (%)	Recurred ≤ 5 Years (%)
Extremities	64	54.7%	78.1%
Other sites	94	61.3%	82.8%

**Table 2:** Assessment of patient-specific tumor characteristics and treatment aspects between patients with a comprehensive care pathway versus fragmented care pathway and early local recurrence versus late local recurrence.

Characteristics	Overall (%)	CCP (%)	FCP (%)	*p*-Value	ELR	LLR	*p*-Value
*N*, (%) Female	158 (100) 74 (46.8)	96 (100) 43 (44.8)	62 (100) 31 (50.0)	0.52	53 (100) 27 (50.9)	105 (100) 47 (44.8)	0.46
Compartment DST-S SST-S Bone	158 (100) 17 (10.8) 124 (78.4) 17 (10.8)	96 (100) 5 (5.2) 77 (80.2) 14 (14.6)	62 (100) 12 (19.5) 47 (75.8) 3 (4.7)	0.005	53 (100) 8 (15.1) 38 (71.7) 7 (13.2)	105 (100) 9 (8.6) 86 (81.9) 10 (9.5)	0.32
Institution 1 2	158 (100) 72 (45.6) 86 (54.4)	96 (100) 39 (40.6) 57 (59.4)	62 (100) 33 (53.2) 29 (46.8)	0.12	53 (100) 23 (43.4) 30 (56.6)	105 (100) 49 (46.7) 56 (53.3)	0.69
Region Face, Head, Neck Upper extremity Lower extremity Axial Intra- and retroperitoneal Urogenital, perianal and anal	158 (100) 8 (5.1) 35 (22.2) 29 (18.3) 41 (25.9) 42 (26.6) 3 (1.9)	96 (100) 3 (3.1) 27 (28.1) 14 (14.6) 22 (22.9) 28 (29.2) 2 (2.1)	62 (100) 5 (8.1) 8 (12.9) 15 (24.2) 19 (30.6) 14 (22.6) 1 (1.6)	0.11	53 (100) 4 (7.5) 13 (24.5) 6 (11.3) 18 (34.1) 12 (22.6) 0 (0.0)	105 (100) 4 (3.8) 22 (20.9) 23 (21.9) 23 (21.9) 30 (28.6) 3 (2.9)	0.21
Initial Size 0–50 mm 51–100 mm 101–150 mm >150 mm n/a	156 (100) 31 (19.9) 53 (34.0) 28 (17.0) 44 (28.1) 2	95 (100) 14 (14.8) 33 (34.7) 15 (15.8) 33 (34.7) 1	61 (100) 17 (27.9) 20 (32.8) 13 (21.3) 11 (18.0) 1	0.05	52 (100) 12 (23.1) 18 (34.6) 10 (19.2) 12 (23.1) 1	104 (100) 19 (18.3) 35 (33.6) 18 (17.3) 32 (30.8) 1	0.75
Tumor grading G1 G2 G3 n/a	156 (100) 33 (21.2) 41 (26.3) 82 (52.5) 2	96 (100) 13 (13.5) 24 (25.0) 59 (61.5) 0	60 (100) 20 (33.3) 17 (28.3) 23 (28.4) 2	0.004	53 (100) 6 (11.3) 16 (30.2) 31 (58.5) 0	103 (100) 27 (26.2) 25 (24.3) 51 (49.5) 2	0.09
Resection margin R0 wide margin/R0 R1 marginal margin/R1 R2 intralesional margin/R2 n/a	131 (100) 58 (44.3) 61 (46.6) 12 (9.1) 27	84 (100) 44 (52.4) 37 (44.0) 3 (3.6) 12	47 (100) 14 (29.8) 24 (51.1) 9 (19.1) 15	0.002	50 (100) 19 (38.0) 28 (56.0) 3 (6.0) 3	81 (100) 39 (48.1) 33 (40.7) 9 (11.2) 24	0.21
Whoops Yes	158 (100) 46 (29.1)	96 (100) 4 (4.2)	62 (100) 42 (67.8)	0.001	53 (100) 36 (67.9)	105 (100) 10 (9.5)	0.001
Adjuvant therapy RT alone CT alone Combined RT & CT None	158 (100) 56 (33.4) 7 (4.4) 9 (5.7) 86 (54.5)	96 (100) 38 (39.6) 6 (6.3) 8 (8.3) 44 (45.8)	62 (100) 18 (29.0) 1 (1.6) 1 (1.6) 42 (67.4)	0.02	53 (100) 19 (35.8) 4 (7.5) 3 (5.7) 27 (50.9)	105 (100) 37 (35.2) 3 (2.9) 6 (5.7) 59 (56.2)	0.59
Time of LR ELR LLR	158 (100) 53 (33.5) 105 (66.5)	86 (100) 27 (31.4) 59 (68.6)	72 (100) 26 (36.1) 46 (63.9)	0.05	-	-	-
Metastases at diagnosis	13 (8.2)	8 (8.3)	5 (8.1)	0.03	8 (15.1)	5 (4.8)	0.02
Status Alive Dead n/a	157 (100) 101 (64.3) 56 (35.7) 1	96 (100) 58 (60.4) 38 (39.6) 0	61 (100) 43 (70.5) 18 (29.5) 1	0.006	52 (100) 25 (48.1) 27 (51.9) 1	105 (100) 76 (72.4) 29 (27.6) 0	0.002

Baseline demographic, anatomical, pathological, surgical, and treatment characteristics of the study cohort, presented for all patients and stratified by care pathway (comprehensive care pathway, CCP; fragmented care pathway, FCP) and by recurrence timing (early local recurrence, ELR; late local recurrence, LLR). Reported variables include sex distribution; anatomical compartment (DST-S, SST-S, BS); treating institution; tumor region; initial tumor size categories; tumor grading (G1–G3); resection margin status (R0, R1, R2); occurrence of unplanned (“whoops”) resections; and receipt of adjuvant radiotherapy, chemotherapy, or combined therapy. The table also details recurrence timing (ELR vs. LLR), metastatic status at diagnosis, and survival status at last follow-up. Values are presented as *N* (%). Group comparisons were performed using the Chi-square test or Fisher’s exact test, depending on expected cell counts, with corresponding *p*-values reported for each characteristic. Abbreviations: CCP, comprehensive care pathway; FCP, fragmented care pathway; ELR, early local recurrence; LLR, late local recurrence; DST-S, deep soft-tissue sarcoma; SST-S, superficial soft-tissue sarcoma; BS, bone sarcoma; RT, radiotherapy; CT, chemotherapy.

**Table 3:** Univariable Cox proportional hazards analysis of factors associated with early local recurrence. The table reports hazard ratios (HR), 95% confidence intervals (CI), and *p*-values for institutional treatment site, tumor grade, anatomical localization, occurrence of unplanned (“whoops”) resections, tumor size category, resection margin status (R0/R1/R2), and care pathway (CCP vs. FCP).

Variable	HR	95%-CI	*p*-Value
Institution (1 and 2)	1.11	0.65–1.92	0.698
Grading (G1, G2 and G3)	1.40	0.98–2.01	0.066
Localisation (Extremity vs. Rest)	1.32	0.75–2.32	0.328
Whoops (Yes vs. No)	1.14	0.64–2.03	0.653
Tumor_Size	0.90	0.70–1.16	0.425
R-Status (R0/R1/R2)	1.03	0.68–1.56	0.879
Care-Pathway (CCP vs. FCP)	1.58	0.92–2.71	0.097

**Table 4:** Restricted multivariable Cox proportional hazards model evaluating independent predictors of early local recurrence. The model includes tumor grading (G1–G3) and care pathway (CCP vs. FCP), selected based on clinical relevance and univariable screening. Hazard ratios (HR), 95% confidence intervals (CI), and *p*-values are reported.

Variable	HR	95%-CI	*p*-Value
Grading (G1, G2, G3)	1.59	1.09–2.31	0.0149
Care-Pathway (CCP, FCP)	2.00	1.14–3.51	0.0150

**Table 5:** Multivariable Firth-penalized logistic regression identifying independent predictors of early local recurrence. Adjusted odds ratios (OR), 95% confidence intervals (CI), *p*-values, and brief interpretative notes are presented for each variable.

Variable	Adjusted OR	95% CI	*p*-Value	Interpretation
Pathway (FCP vs. CCP)	2.83	1.09–6.94	0.031	Fragmented pathway independently increases ELR risk
Tumor grade (per category, G1 → G2 → G3)	1.45	0.89–2.34	0.13	Higher grade trends toward higher ELR
Tumor size (>100 mm vs. ≤100 mm)	1.72	0.78–3.80	0.17	Larger tumors show trend to early relapse
Compartment (DST vs. SST vs. Bone)	1.61	0.68–3.83	0.28	No independent effect after adjustment
Whoops (yes vs. no)	2.34	1.02–5.42	0.046	Unplanned excision more than doubles risk of ELR
Margin (R1/R2 vs. R0)	3.12	1.21–8.04	0.019	Incomplete resection strongly predicts ELR
Adjuvant Therapy (yes vs. no)	0.68	0.30–1.57	0.36	No significant protective effect after adjustment

**Table 6:** Comparison of key surgical quality indicators and adjuvant treatment use between patients treated within the comprehensive care pathway (CCP) and those managed in the fragmented care pathway (FCP). The table reports proportions, standardized mean differences (SMD), and *p*-values for the occurrence of unplanned (“whoops”) procedures, incomplete surgical margins (R1/R2), and receipt of any adjuvant therapy.

Variable	CCP (*n* = 96)	FCP (*n* = 62)	Standardized Difference (SMD)	*p*-Value
Whoops procedure (Yes)	4.2%	68.9%	−1.42	<0.001
Margin status (R1/R2)	47.6%	70.2%	−0.45	0.002
Any adjuvant therapy (Yes)	54.2%	32.8%	+0.43	0.020

## Data Availability

The data presented in this study are available on request from the corresponding author.

## Supplementary Materials

The following supporting information can be downloaded at: https://www.mdpi.com/article/10.3390/cancers18030387/s1, Figure S1: Conceptual overview of the pathway–quality mechanism underlying early local recurrence (ELR) in soft-tissue sarcoma.

## References

[1] Burningham Z., Hashibe M., Spector L., Schiffman J.D. (2012). The Epidemiology of Sarcoma. Clin. Sarcoma Res..

[2] Ducimetière F., Lurkin A., Ranchère-Vince D., Decouvelaere A.-V., Péoc’h M., Istier L., Chalabreysse P., Muller C., Alberti L., Bringuier P.-P. (2011). Incidence of Sarcoma Histotypes and Molecular Subtypes in a Prospective Epidemiological Study with Central Pathology Review and Molecular Testing. PLoS ONE.

[3] Brouns F., Stas M., De Wever I. (2003). Delay in Diagnosis of Soft Tissue Sarcomas. Eur. J. Surg. Oncol..

[4] Gerrand C.H., Wunder J.S., Kandel R.A., O’Sullivan B., Catton C.N., Bell R.S., Griffin A.M., Davis A.M. (2001). Classification of positive margins after resection of soft-tissue sarcoma of the limb predicts the risk of local recurrence. J. Bone Jt. Surgery. Br. Vol..

[5] Nakamura T., Hasegawa M. (2025). Unplanned Excision in Soft Tissue Sarcoma: Current Knowledge and Remaining Gaps. Diagnostics.

[6] Gronchi A., Miah A.B., Dei Tos A.P., Abecassis N., Bajpai J., Bauer S., Biagini R., Bielack S., Blay J.Y., Bolle S. (2021). Soft Tissue and Visceral Sarcomas: ESMO-EURACAN-GENTURIS Clinical Practice Guidelines for Diagnosis, Treatment and Follow-Up☆. Ann. Oncol..

[7] von Mehren M., Kane J.M., Agulnik M., Bui M.M., Carr-Ascher J., Choy E., Connelly M., Dry S., Ganjoo K.N., Gonzalez R.J. (2022). Soft Tissue Sarcoma, Version 2.2022, NCCN Clinical Practice Guidelines in Oncology. J. Natl. Compr. Cancer Netw..

[8] Blay J.Y., Penel N., Valentin T., Anract P., Duffaud F., Dufresne A., Verret B., Cordoba A., Italiano A., Brahmi M. (2024). Improved Nationwide Survival of Sarcoma Patients with a Network of Reference Centers. Ann. Oncol..

[9] Fuchs B., Gronchi A. (2024). Beyond the Sarcoma Center: Establishing the Sarcoma HASM Network-a Hub and Spoke Model Network for Global Integrated and Precision Care. ESMO Open.

[10] Johnson G.D., Smith G., Dramis A., Grimer R.J. (2008). Delays in Referral of Soft Tissue Sarcomas. Sarcoma.

[11] Schärer M., Hösli P., Heesen P., Schelling G., Obergfell T., Nydegger K.N., Studer G., Bode-Lesniewska B., Fuchs B. (2024). Swiss Sarcoma Network Integrated Care in Specialized Networks: Leveraging Early Referrals to Reduce Local Recurrence in Soft Tissue Sarcoma. Cancers.

[12] de Bree E., Michelakis D., Heretis I., Kontopodis N., Spanakis K., Lagoudaki E., Tolia M., Zografakis-Sfakianakis M., Ioannou C., Mavroudis D. (2023). Retroperitoneal Soft Tissue Sarcoma: Emerging Therapeutic Strategies. Cancers.

[13] Gundle K.R., Kafchinski L., Gupta S., Griffin A.M., Dickson B.C., Chung P.W., Catton C.N., O’Sullivan B., Wunder J.S., Ferguson P.C. (2018). Analysis of Margin Classification Systems for Assessing the Risk of Local Recurrence After Soft Tissue Sarcoma Resection. J. Clin. Oncol..

[14] Fujiwara T., Stevenson J., Parry M., Tsuda Y., Kaneuchi Y., Jeys L. (2021). The Adequacy of Resection Margin for Non-Infiltrative Soft-Tissue Sarcomas. Eur. J. Surg. Oncol..

[15] Lemma J., Jäämaa S., Repo J.P., Santti K., Salo J., Blomqvist C.P., Sampo M.M. (2023). Local Relapse of Soft Tissue Sarcoma of the Extremities or Trunk Wall Operated on with Wide Margins without Radiation Therapy. BJS Open.

[16] Hasan O., Nasir M., Jessar M., Hashimi M., An Q., Miller B.J. (2021). Is Local Recurrence in Bone and Soft Tissue Sarcomas Just a Local Recurrence or Does It Impact the Overall Survival, Retrospective Cohort from a Sarcoma Referral Center. J. Surg. Oncol..

[17] Heesen P., Schelling G., Birbaumer M., Jäger R., Bode B., Studer G., Fuchs B. (2024). Swiss Sarcoma Network Real-World-Time Data and RCT Synergy: Advancing Personalized Medicine and Sarcoma Care through Digital Innovation. Cancers.

[18] Fuchs B., Bode B., Heesen P., Kopf B., Michelitsch C., Odermatt M., Giovanoli P., Breitenstein S., Schneider P., Schüpfer G. (2024). Transdisciplinary Sarcoma Care: A Model for Sustainable Healthcare Transformation. Swiss Med. Wkly..

[19] Murphy J., Resch E.E., Leland C., Meyer C.F., Llosa N.J., Gross J.M., Pratilas C.A. (2024). Clinical Outcomes of Patients with CIC-Rearranged Sarcoma: A Single Institution Retrospective Analysis. J. Cancer Res. Clin. Oncol..

[20] Callegaro D., Miceli R., Bonvalot S., Ferguson P.C., Strauss D.C., van Praag V.V.M., Levy A., Griffin A.M., Hayes A.J., Stacchiotti S. (2019). Development and External Validation of a Dynamic Prognostic Nomogram for Primary Extremity Soft Tissue Sarcoma Survivors. EClinicalMedicine.

[21] Heesen P., Studer G., Bode B., Windegger H., Staeheli B., Aliu P., Martin-Broto J., Gronchi A., Blay J.-Y., Le Cesne A. (2022). Quality of Sarcoma Care: Longitudinal Real-Time Assessment and Evidence Analytics of Quality Indicators. Cancers.

[22] Grimer R.J. (2006). Size Matters for Sarcomas!. Ann. R. Coll. Surg. Engl..

[23] Enneking W.F., Spanier S.S., Goodman M.A. (1980). A System for the Surgical Staging of Musculoskeletal Sarcoma. Clin. Orthop. Relat. Res..

[24] Angervall L., Kindblom L.G. (1993). Principles for Pathologic-Anatomic Diagnosis and Classification of Soft-Tissue Sarcomas. Clin. Orthop. Relat. Res..

[25] Larios F., Gonzalez M.R., Ruiz-Arellanos K., Aquilino E Silva G., Pretell-Mazzini J. (2024). Is Unplanned Excision of Soft Tissue Sarcomas Associated with Worse Oncological Outcomes?—A Systematic Review and Meta-Analysis. Cancers.

[26] Melis A.S., Vos M., Schuurman M.S., van Dalen T., van Houdt W.J., van der Hage J.A., Schrage Y.M., Been L.B., Bonenkamp J.B., Bemelmans M.H.A. (2022). Incidence of Unplanned Excisions of Soft Tissue Sarcomas in the Netherlands: A Population-Based Study. Eur. J. Surg. Oncol..

[27] von Konow A., Ghanei I., Styring E., Vult von Steyern F. (2021). Late Local Recurrence and Metastasis in Soft Tissue Sarcoma of the Extremities and Trunk Wall: Better Outcome After Treatment of Late Events Compared with Early. Ann. Surg. Oncol..

[28] Pisters P.W., Leung D.H., Woodruff J., Shi W., Brennan M.F. (1996). Analysis of Prognostic Factors in 1,041 Patients with Localized Soft Tissue Sarcomas of the Extremities. J. Clin. Oncol..

[29] Serban B., Cretu B., Cursaru A., Nitipir C., Orlov-Slavu C., Cirstoiu C. (2023). Local Recurrence Management of Extremity Soft Tissue Sarcoma. EFORT Open Rev..

[30] Traub F., Griffin A.M., Wunder J.S., Ferguson P.C. (2018). Influence of Unplanned Excisions on the Outcomes of Patients with Stage III Extremity Soft-Tissue Sarcoma. Cancer.

[31] Jubane M., Rennick A.C., Villavicencio J.J., Ferreira de Souza F., Peters V., Jonczak E., Bialick S., Dhir A., Grossman J., Trent J.C. (2024). Imaging-Based Disease Assessment and Management Recommendations: Impact of Multidisciplinary Sarcoma Tumor Board. Cancers.

