# Supplementary figures and images for: Organizing Care Matters: Fragmented Pathways Double Early Local Recurrence Risk in Sarcoma
Source: Cancers (Basel). 2026 Jan 27;18(3):387. doi: 10.3390/cancers18030387 (PMC12896758; doi:10.3390/cancers18030387)

Supplementary

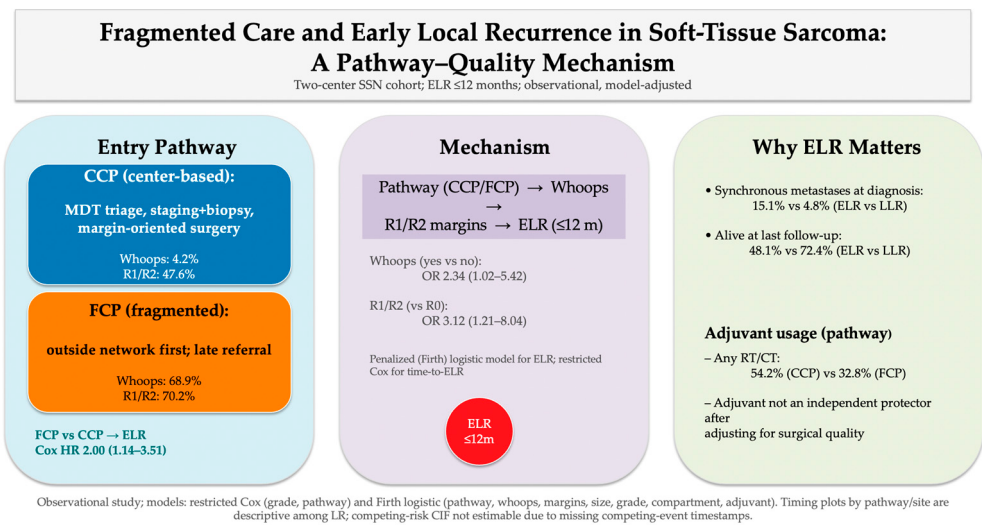

Supplement: Supplementary file 1 [file cancers-18-00387-s001.zip › cancers-4100844-supplementary.pdf]
